# Adverse immunological responses against non-viral nanoparticle (NP) delivery systems in the lung

**DOI:** 10.1080/1547691X.2021.1902432

**Published:** 2021-05-06

**Authors:** Leonor de Braganca, G. John Ferguson, Jose Luis Santos, Jeremy P. Derrick

**Affiliations:** a Lydia Becker Institute of Immunology and Inflammation, School of Biological Sciences, Faculty of Biology, Medicine and Health, Manchester Academic Health Science Centre, The University of Manchester, Manchester, UK; b Translational Science and Experimental Medicine, Research and Early Development, Respiratory and Immunology, BioPharmaceuticals R&D, AstraZeneca, Cambridge, UK; c Dosage Form Design Development, BioPharmaceuticals Development, R&D, AstraZeneca, Cambridge, UK

**Keywords:** Nanoparticles, gene therapy, lung, inhalation, immune response

## Abstract

There is a large, unmet medical need to treat chronic obstructive pulmonary disease, asthma, idiopathic pulmonary fibrosis and other respiratory diseases. New modalities are being developed, including gene therapy which treats the disease at the DNA/RNA level. Despite recent innovations in non-viral gene therapy delivery for chronic respiratory diseases, unwanted or adverse interactions with immune cells, particularly macrophages, can limit drug efficacy. This review will examine the relationship between the design and fabrication of non-viral nucleic acid nanoparticle (NP) delivery systems and their ability to trigger unwanted immunogenic responses in lung tissues. NP formulated with peptides, lipids, synthetic and natural polymers provide a robust means of delivering the genetic cargos to the desired cells. However NP, or their components, may trigger local responses such as cell damage, edema, inflammation, and complement activation. These effects may be acute short-term reactions or chronic long-term effects like fibrosis, increased susceptibility to diseases, autoimmune disorders, and even cancer. This review examines the relationship between physicochemical properties, i.e. shape, charge, hydrophobicity, composition and stiffness, and interactions of NP with pulmonary immune cells. Inhalation is the ideal route of administration for direct delivery but inhaled NP encounter innate immune cells, such as alveolar macrophages (AM) and dendritic cells (DC), that perceive them as harmful foreign material, interfere with gene delivery to target cells, and can induce undesirable side effects. Recommendations for fabrication and formulation of gene therapies to avoid adverse immunological responses are given. These include fine tuning physicochemical properties, functionalization of the surface of NP to actively target diseased pulmonary cells and employing biomimetics to increase immunotolerance.

## Introduction

Chronic pulmonary diseases, such as chronic obstructive pulmonary disease (COPD), asthma and idiopathic pulmonary fibrosis (IPF), compromise the anatomy and change the physiology of the lung. COPD is characterized by chronic lung inflammation, mucus hypersecretion and impaired lung function, resulting in permanent airflow constraints (Vlahos and Bozinovski 2014; Barnes et al. 2015; Belchamber and Donnelly 2017; Jubrail et al. 2017; Yamasaki and Eeden 2018; Agustí and Hogg 2019; Celli and Wedzicha 2019). Inhaled irritants trigger inflammatory responses that progressively narrow the airways and destroy the alveoli, leading to the development of chronic respiratory symptoms like coughing, sputum production and breathing difficulties (Laouini et al. 2014; Vlahos and Bozinovski 2014; Barnes et al. 2015; Yamasaki and Eeden 2018; Agustí and Hogg 2019; Celli and Wedzicha 2019). Asthma is a chronic respiratory disease induced by allergens also characterized by breathing difficulties, wheezing and coughing due to infiltration of inflammatory cells, pulmonary wall thickening and airway obstruction from mucus hypersecretion (Costa et al. 2015; Choi et al. 2017; Lambrecht et al. 2017). The hallmarks of asthma in atopic individuals are the T-helper Type 2 (T_H_2) cell immune responses (Lambrecht and Hammad 2014; Lambrecht et al. 2017, 2019). IPF is an incurable progressive interstitial lung disease that involves the accumulation of pro-fibrotic macrophages and fibroblasts that induce inflammation and fibrosis due to excessive collagen deposition; patients eventually die as the irreversibly-changed lung architecture disrupts gas exchange leading to respiratory failure (Wuyts et al. 2013; Stahl et al. 2013; Costa et al. 2015; Grabiec and Hussell 2016; Kolahian et al. 2016; Garbuzenko et al. 2017; Heukels et al. 2019; Li et al. 2019).

## Value and opportunities for gene therapy

Current strategies for treating COPD include bronchodilators (e.g. salbutamol), steroid inhalers such as budesonide, or mucolytics like carbocisteine and acetylcysteine (Barnes and Stockley 2005). Example drugs for treating asthma include the preventer inhaler beclometasone, the reliever inhaler terbutaline and monoclonal antibody therapies such as benralizumab and omalizumab (Green et al. 2003). However, there is an urgent need for new therapies to address chronic respiratory diseases, as current treatments are limited to mitigating the symptoms, rather than tackling the root cause of the disease in question. Gene therapy has recently gained considerable attention as a therapeutic modality, due to its ability to correct any post-transcriptional gene mutation (Schlosser et al. 2018). DNA and RNA molecules can be used in various ways to target and modulate gene expression for therapeutic benefit.

The current development status of candidate gene therapies for chronic respiratory diseases is summarized in Table 1. Work in this area is current and ongoing: many of these developments have been reported relatively recently and it is likely that more will be forth-coming in the near future. The question of lung immunogenicity will become increasingly important as some of these therapies are taken into clinical trials. In the context of the lung, alveolar macrophages (AM) can stimulate innate and dendritic cells (DC) adaptive immune responses which increase clearance of foreign inhaled material and may activate pro-inflammatory immune responses. While these physiological responses are protective when the lung is under threat, they are undesirable for therapy: not only can they aggravate the diseased state of the lung but they also intervene in the delivery of the therapeutic gene to the target cell/tissue. The safety of gene delivery systems and their interaction with host cells therefore needs to be investigated at some point during clinical development.

**Table 1.** Current candidate gene therapies for COPD, asthma, and IPF.

Disease	Gene therapy approach	Outcome of intervention	References
COPD	Aerosol delivery of cationic liposome complexed to plasmid encoding recombinant human α1-anti-trypsin gene to New Zealand White rabbits	Transfection and expression of human α1-antitrypsin in alveolar epithelial cells for 7 d after single dose	(Canonico et al. 1994)
	Transfection of human bronchial epithelial cells with p65 siRNA and treatment with NF-κB small molecule inhibitor	Attenuation of NF-κB pathway Suppression of pro-inflammatory cytokine IL-1β- and IL-17A-induced expression of mucin *(MUC)5AC* gene	(Fujisawa et al. 2009)
	Treatment of human alveolar epithelial lung cells with cationic polymeric NP attached to miR-146a	Inhibited expression of IL-1 receptor-associated kinase (*IRAK1*) genes Reduced IL-1β-dependent activation of NF-κB	(Mohamed et al. 2019)
Asthma	Intratracheal instillation of dexamethasone attached to cationic polymeric NP loaded with Vitamin D binding protein (VDBP) to OVA-sensitized mice model of allergic asthma	Down-regulated alveolar macrophage VDBP expression Reduced airway inflammation Decreased mucus hypersecretion Expression of T_H_2 cytokines	(Choi et al. 2017)
	Intranasal delivery of recombinant vaccinia vector encoding T_H_1 cytokine IL-12 to OVA-sensitized mice	Inhibited local Type 2 cytokine production Suppressed allergy and airway hyper-reactivity mediated by boosting endogenous IFNγ expression Reversed suppression of local cell-mediated immunity	(Hogan et al. 1998)
	Intranasal delivery of cationic chitosan NP loaded with IFNγ pDNA to OVA-sensitized mice	Reduced airway hyper-responsiveness to methacholine Reduced IFNγ production by OVA-sensitized CD8^+^ T-cells Decreased DC activity	(Kumar et al. 2003; Kong et al. 2008)
	Intravenous delivery of IFNγ pDNA to OVA-sensitized mice	Decrease in T_H_2 immune response Reduced IFNγ production by reduction in DC activity and OVA-sensitized CD8^+^ T-cell activation	(Nakagome et al. 2009)
IPF	Inhaled delivery of cationic nanostructure lipid carriers loaded with prostaglandin E (PGE2) and ECM-degrading enzyme MMP3, CCL12 and hypoxia-inducible factor 1α (HIF1A) siRNA to mice model of IPF	Reduced expression and production of MMP3, CCL12 and HIF1A Decreased lung inflammation and fibrotic tissue damage Down-regulated expression of connective tissue growth factor (*CTGF*), *TGFB*, and *TGFBR* genes Reduced proliferation, activation and migration of fibroblasts Decreased collagen production and secretion by fibroblasts	(Garbuzenko et al. 2017)

### Gene delivery systems

Different types of DNA and RNA molecules, either unmodified or chemically modified, have been developed for use in gene therapy. The advantages of using RNA over DNA for gene therapy are low immunogenicity, an inability to integrate or mutate the genome, absence of nuclear transport, and the ability to “pharmaco-evolve”: RNA-based therapeutics can change their targeting sequence as the disease evolves, allowing it to keep up with and to treat a progressing disease (Kormann et al. 2011; Yin et al. 2014; Dowdy 2017). A summary of different gene therapy modalities is presented in Table 2.

**Table 2.** Gene therapy modalities used in disease models.

Gene therapy approach	Disease model	Outcome of intervention	References
*mRNA*	Congenital surfactant protein B (SP-B) deficiency	Decreased interaction with TLR3, TLR7, TLR8 and RIG-I Increased stability Low immunogenicity Prolonged gene expression	(Kormann et al. 2011)
*mRNA*	n/a	Increased systemic production of serum protein erythropoietin Increased circulating reticulocytes Increased % of circulating RBC No immunogenicity Does not interfere with potency of regulatory sequences	(Thess et al. 2015)
*mRNA*	n/a	Leukotriene inhibitors for lung cancer treatment increase transfection efficacy by 200%	(Patel et al. 2017)
*siRNA*	Hereditary transthyretin-mediated amyloidosis	Reduced production of misfolded transthyretin protein in the liver Slow progression of hereditary transthyretin-mediated amyloidosis	(Kristen et al. 2019)
*siRNA*	Multi-drug resistant lung, breast, colon, and ovarian cancer	Suppress genes of pump drug resistance (drug efflux) Suppress genes of non-pump resistance (anti-apoptosis) Apoptosis induction in cancer cells Increased toxicity of anti-cancer drug to cancer cells Near complete tumor regression	(Saad et al. 2008; Taratula et al. 2013)
*DNA*	Lung cancer	Increased anti-cancer drug toxicity Passive and active tumor-targeting ability Increased *in vivo* gene transfection efficiency Enhanced anti-tumor activity	(Shao et al. 2015)
*ASO*	Drug-sensitive and multi-drug resistance small-cell lung cancer	Suppress genes of pump drug resistance (drug efflux) Suppress genes of non-pump resistance (anti-apoptosis) Enhanced apoptosis induction in cancer cells Increased anti-cancer drug toxicity Significant tumor regression	(Garbuzenko et al. 2010; Pakunlu et al. 2004)

DNA and RNA molecules cannot freely diffuse across cell membranes, due to their large size and negative charge; they therefore need a carrier to mediate entry into target cells and prevent stimulation of an immune response (Yin et al. 2014; Cullis and Hope 2017; Thanki et al. 2018). Nanoparticles (NP) are robust non-viral vectors that can be formulated using different materials such as peptides, synthetic or natural polymers, and lipids with different physicochemical characteristics which protect nucleic acids from enzymatic degradation. Compared to viral vectors, these non-viral counterparts are safer, easier to produce, cost-efficient, easily functionalized to improve transfection and can encapsulate nucleic acids of virtually any size (Chan et al. 2012; Zhao and Huang 2014; Pozo-Rodríguez et al. 2016). In addition, the delivery system also requires a mechanism to ensure endosomal escape: the materials used to deliver nucleic acids should therefore have endosomolytic properties to prevent degradation of the genetic material inside the lysosomes (Wittrup et al. 2015; Bus et al. 2018; Yanez Arteta et al. 2018).

### Cationic lipids

Cationic lipids overcome natural barriers in gene therapy such as the extracellular environment, the cell membrane, the intracellular environment and the endosome by taking advantage of their positive charge to associate with negatively-charged cell components. Genetic material is packaged and condensed into lipid nanoparticles (LNP) for delivery via the electrostatic interaction between the positively-charged lipid head group and the negatively-charged phosphate backbone of the nucleic acid (Jensen et al. 2012; Levine et al. 2013; Rehman et al. 2013; Dan and Danino 2014; Pozo-Rodríguez et al. 2016; Lin et al. 2018; Thanki et al. 2018). This interaction should be strong enough to protect the genetic material from enzymatic degradation and immune recognition in the extracellular and intracellular environment but should be weak enough for successful release of the genetic material inside the endosome (Zhao and Huang 2014; Pozo-Rodríguez et al. 2016). Pairing between the cationic lipid head group of the NP and the anionic phospholipid of the endosomal membrane neutralizes the particle charge, resulting in disassociation of the nucleic acid, endosomal membrane destabilization, and release of nucleic acid into the cytosol (Hafez et al. 2001; Gilleron et al. 2013; Rehman et al. 2013; Zhao and Huang 2014; Yanez Arteta et al. 2018).

The cationic lipid GL-67 has been used as a non-viral vector to treat cystic fibrosis (CF), which is caused by mutations in the CF transmembrane conductance regulator (*CFTR*) gene (Alton et al. 1999; He et al. 2019). GL-67 was complexed to the *CFTR* gene and delivered to the lungs *via* nebulization and intranasal administration, resulting in ∼25% restoration of the chloride channel function in the lungs of CF patients (Alton et al. 1999). However, the lipid-DNA complex induced a minor pro-inflammatory response, possibly due to internalization of the complex by AM (Alton et al. 1999; Merkel and Kissel 2012).

Cationic solid lipid nanoparticles (cSLN) are composed of biodegradable, biocompatible lipids and surfactants but have been shown to strongly interact with the cell membrane of human neutrophils, causing membrane lysis (Hwang et al. 2015). Consequently, calcium ion (Ca^2+^) mobilization triggered by the compromised membrane, induces respiratory burst and degranulation wherein elastase and neutrophil extracellular traps (NET) are released into the extracellular environment (Hwang et al. 2015). While beneficial against invading pathogens, extensive activation of neutrophils and excess oxidative stress can be harmful to host cells. Treatment of human alveolar epithelial A549 cells with cationic lipid Oligofectamine (OF) NP loaded with DNA up-regulated expression of genes involved in apoptosis and oxidative stress, and down-regulated genes related to cell growth, maintenance and proliferation, which is deleterious to the cell (Omidi et al. 2008). Rather than inducing the intended therapeutic changes in gene expression driven by the encapsulated DNA, off-target changes in gene expression also occurred.

To overcome the cytotoxicity of positive surface charges and to increase transfection efficiency, ionizable cationic lipids have been used instead of those with a permanent charge. Alnylam Pharmaceuticals manufactured Patisiran, the first RNAi therapeutic loaded in a LNP approved by the FDA and EMA for the treatment of hereditary transthyretin-mediated amyloidosis which causes neuropathy (Jorge et al. 2019; Kristen et al. 2019). Patisiran is formulated with the ionizable cationic lipid DLin-MC3-DMA (MC3) (pK_a_ = 6.44), the most potent ionizable cationic lipid for siRNA delivery. LNP-RNA systems formulated with this lipid have an encapsulation efficiency close to 100%, very low median effective dose (ED_50_), increased RNA delivery, increased protein production, and greater therapeutic effects (Semple et al. 2010; Belliveau et al. 2012; Jayaraman et al. 2012; Kulkarni et al. 2018; Cullis and Hope 2017; Maugeri et al. 2019).

Ionizable cationic lipids have functional groups with pK_a_ values < 7: at acidic pH, they become positively-charged and can efficiently encapsulate negatively-charged molecules into the NP. However, at physiological pH, they adopt a relatively neutral surface charge that overcomes the toxicity associated with permanently charged cationic lipids. Inside endosomes with acidic pH, ionizable cationic lipids become positively-charged and interact with the anionic lipids of the endosomal membrane to promote release of its cargo into the cytosol (Semple et al. 2010; Jayaraman et al. 2012; Cullis and Hope 2017; Kulkarni et al. 2018; Yanez Arteta et al. 2018; Buck et al. 2019). Robinson et al. (2018) formulated LNP with MC3, encapsulating chemically-modified *CFTR* mRNA, administered the NP *in vitro* to patient-delivered bronchial epithelial cells and intranasally to *CFTR* gene-knockout mice. As a result, there was an increase in local-ization of the CFTR protein in the plasma membrane of bronchial epithelial cells and long-lasting restoration of chloride channel function by one third in airway epithelial cells.

### Peptides

Peptides formulated into delivery systems typically consist of an anchor moiety which interacts with the hydrophobic regions of other components, a cationic moiety that electrostatically interacts with the negatively-charged nucleic acid and a targeting moiety which directs the vector to a specific cell type. The peptide, formulating materials and the nucleic acid spontaneously self-assemble to form a binary complex which can therefore act as a non-viral vector (Guan et al. 2019). These peptides can also be used to functionalize other delivery systems, through the targeting moiety, to mitigate off-target effects and to direct delivery to specific cells (Martin and Rice 2007; Rodríguez Gascón et al. 2013). The transfection efficiency of non-viral vectors can be enhanced by adding pH-sensitive peptides with fusogenic properties, such as GALA (Glu-Ala-Leu-Ala) and KALA (Lys-Ala-Leu-Ala) to the formulation; these pH-sensitive peptides disrupt the membrane during endosomal acidification and promote release of the encapsulated molecule into the cytoplasm (Mann et al. 2008; Lam et al. 2012). If non-viral vectors with pH-sensitive peptides are internalized *via* pathways that bypass the lysosome, such as macropinocytosis and caveola-mediated endocytosis, the fusogenic peptides can no longer disrupt the endosomal membrane and the cargo is not released into the cytosol (Loughran et al. 2015).

Cell-penetrating peptides (CPP) synthesized or derived from viral peptides that induce cell entry, have also been used to enhance the transfection efficiency of delivery systems (Lam et al. 2012). Examples include TAT, penetratin, and transportan; they consist of 10–30 positively-charged residues which can electrostatically associate with negatively-charged nucleic acids and penetrate the negatively-charged cell membrane (Merkel et al. 2014; Pozo-Rodríguez et al. 2016). CPP can be internalized through direct translocation, transient pore formation and endocytosis, depending on the nature of the cargo and where on the peptide it is associated (Mann et al. 2008; Mager et al. 2010). However, CPP can only interact nonspecifically with any cell they encounter (McErlean et al. 2015; Chen et al. 2017; Kang et al. 2019).

A difficulty in using peptides as delivery systems is that they are susceptible to rapid degradation by proteases present in biological fluids (Loughran et al. 2015; McErlean et al. 2015; Kang et al. 2019). Peptides used to interact with genetic material are polycationic and are liable to be cytotoxic, causing damage to the cell membrane and even cell death (Fabre and Collins 2006). Furthermore, cationic peptides adsorb anionic molecules in biological fluids, disrupting the electrostatic forces holding peptide/DNA complexes together (Fabre and Collins 2006). Their poor binding to genetic material can result in premature release of the genetic material upon *in vivo* administration (Mann et al. 2008; Kang et al. 2019). For all these reasons, peptide delivery systems exhibit *in vivo* instability (Fabre and Collins 2006; Mann et al. 2008; McErlean et al. 2015; Chen et al. 2017; Kang et al. 2019). However, there is an opportunity to develop hybrid delivery systems composed of peptides and other materials, such as lipids and polymers, to improve nucleic acid delivery by peptide NP.

### Synthetic polymers

Polymers can be designed to adopt properties necessary to perform different functions and are routinely exploited as non-viral vectors with low immunogenicity. Polycationic polymers, such as polyethylenimine (PEI) and chitosan, can interact electrostatically with nucleic acids to assemble into compact polyplexes; the high number of amino groups gives the polymers high positive surface charge densities (Nayerossadat et al. 2012; Bus et al. 2018). Polyplexes are internalized by cells via endocytosis, such as clathrin-mediated endocytosis and micropinocytosis, which dictate the intracellular localization of the polyplex following uptake (Bus et al. 2018).

PEI can induce endosomal escape following cellular uptake, evading degradation of the genetic material inside endolysosomes via the “proton sponge” effect (Tehrani et al. 2007; Lam et al. 2012; Jesus and Zuhorn 2015; Bus et al. 2018). Some studies have supported other theories of endosomal escape by polymers: the interaction of the cationic polyplex with the negatively-charged endosomal membrane causes membrane disruption, local pore formation and the release of the polyplex into the cytosol (Jin et al. 2006; Lam et al. 2012; Rehman et al. 2013; Bus et al. 2018; Jorge et al. 2019). The polycationic nature of PEI and its high molecular weight help enhance its transfection efficiency, at the cost of increasing its cytotoxicity: high cationic charge densities and resistance of PEI to degradation results in intracellular accumulation, which could contribute to immunogenicity (Xu et al. 2008; Cho 2012). Previous studies on PEI as a vaccine adjuvant have shown that these properties make it a potent immunostimulant (Regnstrom et al. 2003; Grant et al. 2012; Sheppard et al. 2014; Shen et al. 2017). PEI has been crosslinked by biodegradable disulfide and ester linkages, to increase its biocompatibility, degradability and safety, and to aid in the release of genetic material from the polyplex without modifying its high transfection efficiency (Cho 2012).

Unlike PEI, chitosan is a naturally occurring, biodegradable polymer that is biocompatible and has low toxicity (Cho 2012). Its muco-adhesive and muco-penetrative properties make chitosan a good candidate for pulmonary administration but, unlike PEI, chitosan is poor at promoting endosomal escape and therefore has low transfection efficiency (Jin et al. 2006; Cho 2012; Lam et al. 2012). Jin et al. (2006) modified chitosan with urocanic acid to promote release of the nucleic acid payload into the cytosol and improve endosomal escape via the “proton sponge” effect. When complexed with the programmed cell death protein 4 (*PDCD4*) tumor suppressor gene and aerosolized into the lungs of model lung cancer mice, apoptosis was enhanced, the cell cycle arrested and tumor angiogenesis was suppressed.

Alternatively, hyperbranched poly-(β-amino esters; PBAE) with degradable backbones have been used as vectors for gene delivery because they maintain positive charges at physiological pH, reducing their toxicity and increasing their stability (Patel et al. 2019). Electrostatic interaction between hyperbranched PBAE and mRNA results in encapsulation of the nucleic acid within the polyplex. Patel et al. (2019) nebulized a polyplex loaded with luciferase mRNA into the lungs of Ai14 Cre reporter mice, resulting in transfection of 24.6% of all lung epithelial cells but the transient nature of gene expression suggested that more frequent doses were required. Mastorakos et al. (2015) formulated highly-stable pDNA NP with mucus-penetrating properties, composed of the biocompatible and biodegradable polymer PBAE densely-coated with polyethylene glycol (PEG). Following intratracheal administration to mouse lungs, the PEG-coated PBAE NP passed through the thick mucus layer to reach and transfect the underlying epithelium (Mastorakos et al. 2015). The transfection efficiency of PBAE NP was superior to that of PEI NP, as seen by the prolonged expression of the delivered gene for a minimum of 4 months.

For optimal delivery, polymeric NP should be cationic, have a low molecular mass, present biodegradable features, and incorporate hydrophobic side chains for stability (Yan et al. 2016). Furthermore, they should be able to withstand the high shear stresses exerted during nebulization (Merkel et al. 2012). An optimal polymeric NP formulation is one which would incorporate the high transfection efficiency and buffering capacity of PEI with the biocompatibility and degradability of chitosan. This could be achieved by grafting PEG or chitosan onto PEI NP, to reduce interaction with soluble components and cells in the lung, maintain stability during nebulization and *in vivo,* and enhance gene delivery (Merkel et al. 2012; Cho 2012).

### Challenges of gene delivery to the lung

Administration of therapeutics by inhalation is an attractive route for noninvasive delivery. It is the easiest and most common way of delivering agents directly to the lungs due to the large surface area of the lung, its high membrane permeability, its extensive vascularization, and low levels of degradative enzymatic activity. This results in delays in drug metabolism causing accumulation, with minimal cytotoxicity and better therapeutic effect at a lower dose (Mansour et al. 2009; Semple et al. 2010; Nanjwade et al. 2011; Ruge et al. 2011; Weber et al. 2014; Bhardwaj et al. 2015; El-Sherbiny et al. 2015; Lee et al. 2015; Moreno-Sastre et al. 2015; Dasa et al. 2017; Ngan and Asmawi 2018). However, delivery to the right area poses challenges due to the complex structure of the lung and potential interactions with the immune system.

### Delivery of NP to the lung

To exert therapeutic effects in the lung, the particles must deposit locally at the site of disease (Ngan and Asmawi 2018) either in the small airways for COPD (Barnes and Stockley 2005) and IPF (Wuyts et al. 2013) or the upper airways for asthma (Borghardt et al. 2018). The aerodynamic diameter of particles dictates where they deposit in the lung (Sung et al. 2007; Weber et al. 2014; El-Sherbiny et al. 2015; Thanki et al. 2018). Small, light particles (aerodynamic diameter < 1 µm) deposit in the alveoli by Brownian diffusion but are more easily exhaled (Sung et al. 2007; Hayes and Bakand 2014; Weber et al. 2014; El-Sherbiny et al. 2015). Large particles (aerodynamic diameter > 5 µm) collide with the pulmonary walls and deposit on bifurcations of the upper airways *via* impaction due to poor response to changes in air stream; particles with aerodynamic diameters between 1 and 5 µm reach the lower airways and deposit slowly on bronchioles and alveoli by sedimentation due to gravity (Sung et al. 2007; Hayes and Bakand 2014; Maretti et al. 2017; Ngan and Asmawi 2018).

Inhalation devices nebulize NP into liquid aerosol droplets or spray-dry NP into inhalable micro-aggregates with diameters between 1.5 and 3.5 µm (Paranjpe and Müller-Goymann 2014; Sosnowski 2015; Borghardt et al. 2018). This ensures distribution of NP throughout the respiratory tract to prevent exhalation of the NP, to promote deposition into the deep lung, to target the NP to the therapeutic site, and to avoid clearance by the mucociliary escalator (Lam et al. 2012; Zhou et al. 2015; Dua et al. 2019). This clearance mechanism, which functions to clear the airways of microbes and particles, is composed of a monolayer of ciliated epithelial cells and non-ciliated goblet cells that secrete mucus (Nanjwade et al. 2011; Ruge et al. 2013; Weber et al. 2014; Costa et al. 2015; El-Sherbiny et al. 2015; Thanki et al. 2018). The impermeable mucus barrier traps the particles, preventing their passage to the lower airways, and the cilia beat in order to sweep the mucus up to be coughed out or swallowed (Paranjpe and Müller-Goymann 2014; El-Sherbiny et al. 2015; Ruge et al. 2013; Mangal et al. 2017; Ngan and Asmawi 2018).

Once inhaled, the agglomerations of micron-sized NP clusters must dissolve into the pulmonary fluid and disperse into individual NP in order to be absorbed (Braakhuis et al. 2014; Borghardt et al. 2018; Strong et al. 2018). Dissolution rates are a double-edged sword, however: although delivery systems with slow dissolution rates exhibit longer residence times favoring lung absorption, they are also at risk of being cleared by intrinsic pulmonary defense mechanisms such as the mucociliary escalator and phagocytosis by AM (Borghardt et al. 2018; Strong et al. 2018). Similarly, increased residence time also leads to increased interaction with the immune system (Geiser 2010; Frohlich and Salar-Behzadi 2014; Hayes and Bakand 2014; Patel et al. 2015; Thanki et al. 2018).

### NP interactions with immune system

Apart from the complex architecture of the lung, inhaled material that enters the lungs may encounter the innate immune system which has evolved to protect the host from pathogens; these mechanisms shorten drug half-life, decrease bioavailability and limit the therapeutic effects of inhaled nanomedicines (Muhlfeld et al. 2008; Paranjpe and Müller-Goymann 2014; El-Sherbiny et al. 2015; Patel et al. 2015; Ruge et al. 2013; Mangal et al. 2017; Ngan and Asmawi 2018). In addition, by internalizing the inhaled drug, not only do innate immune cells in the lung interfere with the delivery of the drug to its target lung region or target cell, but they can also initiate an undesirable immune response. Two cell types that are thought to be important in the recognition of NP are the DC and AM. Immature DC in the lung periphery form tight junctions with pulmonary epithelial cells and extend their dendrites into the lumen of the airways for internalization of foreign material that enter the airways (Lambrecht and Hammad 2012a, 2012b, 2014). The DC become activated, undergo maturation, then travel to the T-cell zone in the mediastinal lymph nodes and present the digested material as a complex with major histocompati-bility complex (MHC) on their surface to prime naïve T-cells (Lambrecht and Hammad 2012b, 2014; Boraschi et al. 2017; Thanki et al. 2018).

AM populate the inner surface of the lung underneath the pulmonary surfactant layer that coats the alveolar epithelium, where the air-liquid barrier lies (Geiser 2010; Hussell and Bell 2014; Patel et al. 2015; Desch et al. 2016). AM are sentinels that maintain pulmonary homeostasis by engulfing debris, dead cells and pathogens, and by catabolizing lung surfactant into lipids and proteins (Lee et al. 2015; Mathie et al. 2015; Belchamber and Donnelly 2017; Garbi and Lambrecht 2017; Allard et al. 2018; Costa et al. 2018; Evren et al. 2020). For every alveolus, there are 8–12 AM that remove the inhaled NP and act to limit the residence time of NP in the lung to only a few hours. This increased turnover would result in suboptimal therapeutic effects and, as a result, NP would have to be administered more frequently, which could result in patient noncompliance (Geiser 2010; El-Sherbiny et al. 2015; Lee et al. 2015; Ngan and Asmawi 2018).

### Immune responses to NP in the lung

High concentrations of NP and prolonged retention in the lung can cause local responses such as irritation, cellular injury, edema and inflammation (Braakhuis et al. 2014; Hayes and Bakand 2014). These effects may be acute short-term reactions or they can prolong into chronic long-term effects like fibrosis, and may even spread to extrapulmonary organs (Geiser 2010; Ruge et al. 2013; Hayes and Bakand 2014; Patel et al. 2015).

The nucleic acid encapsulated in the NP may induce a potent innate immune response by acting as agonists of pattern recognition receptors which sense “non-self” nucleic acids on the endosomal membrane (Szebeni and Moghimi 2009; Zhao and Huang 2014; Cullis and Hope 2017; Thanki et al. 2018). Nonspecific activation of DC occurs either by direct binding of the inhaled NP to pattern recognition receptors (PRR) on their surface, or by indirect mechanisms: binding of inhaled material to PRR on epithelial cells that become activated and secrete DC-activating cytokines (Lambrecht and Hammad 2012a, 2012b, 2014). The association between the inhaled particles and Toll-like receptors (TLR)-1, −2, and −4 can result in the production of pro-inflammatory cytokines, chemokines and growth factors, resulting in infiltration of neutrophils, and oxidative stress from release of reactive oxygen species (ROS) that potentially cause cellular or even organ damage (Merkel et al. 2012; Costa et al. 2015; Galbiati et al. 2016; Thanki et al. 2018).

The lower airways are kept sterile by Type II alveolar pneumocytes that produce lung surfactant containing the proteins SP-A and SP-D (Weber et al. 2014; Costa et al. 2015; Thanki et al. 2018) that opsonize inhaled particles, forming a biocorona (Braakhuis et al. 2014; Boraschi et al. 2017; Konduru et al. 2017). The biocorona modulates composition, size, and surface charge of the NP which could promote off-target effects and their clearance, and affects their deposition and interactions with cells (Walkey et al. 2012; Konduru et al. 2017; Thanki et al. 2018; He et al. 2019). For example, the biocorona can activate the complement system (Boraschi et al. 2017) and can bind to and activate AM (Trapnell and Whitsett 2002; Konduru et al. 2017; Thanki et al. 2018). The coated particles are phagocytized by AM that can destroy them within their phagolysosomes, or they travel up the mucociliary escalator for removal from the lungs (Ruge et al. 2013; Lee et al. 2015; Konduru et al. 2017; Mangal et al. 2017; Ngan and Asmawi 2018). While NP generally have a size < 1 µm, they can agglomerate post-delivery into the lungs, creating micron-sized clusters. The optimal size for AM to ingest material via phagocytosis is 1–6 µm (Hirota and Terada 2012; Lee et al. 2015; Ngan and Asmawi 2018). As this overlaps with the ideal size for particle deposition, this poses a problem for NP therapeutics.

### Impact of charge, composition, size, and/or shape on interaction with pulmonary cells

Inadvertent interactions between NP and immune cells may trigger signaling pathways that induce inflammation and complement activation which can, through unwanted particle uptake, develop into harmful responses that may increase susceptibility to diseases, autoimmune disorders and even cancer (Kononenko et al. 2015; Patel et al. 2015; Liu et al. 2017; Fadeel 2019). The immune system can react to NP in a similar manner to pathogens. The immune responses that NP elicit depend on their physicochemical properties such as size, charge, shape, hydrophobicity, and stiffness (Liu et al. 2017; Muhammad et al. 2020). To be able to re-design NP formulations to prevent adverse immune responses, one must first understand how NP interact with cells of the immune system and how different physicochemical properties affect interaction. A summary of recent findings in this area is presented in Table 3.

**Table 3.** Effect of physicochemical properties of NP on the immune system.

Physicochemical property	Effects	References
Size	Dictates endocytic uptake route and subcellular localization Smaller NP have higher surface-to-volume ratios and higher charge densities therefore induce stronger pro-inflammatory responses, potentially more cytotoxic Larger NP are retained in lung, likely to be internalized by macrophages Smaller NP can enter the systemic circulation through the lung, likely to be internalized by DC	(Dobrovolskaia et al. 2016; Hoshyar et al. 2016; Liu et al. 2017; Muhammad et al. 2020; Thorp et al. 2020)
Shape and stiffness	Spherical shapes are more rapidly and efficiently taken up by macrophages than non-spherical shapes (fibre, worm-like, rod, cylinder) Rigid NP cause damage to cell membrane during internalization	(Champion and Mitragotri 2009; Patel et al. 2015; Thorp et al. 2020)
Charge	Positive surface charges enhance internalization by macrophages and DC due to electrostatic interaction with negatively-charged cell membrane High surface charge densities, either too negatively- or positively-charged, enhance cellular uptake	(Patel et al. 2015; Dobrovolskaia et al. 2016; Liu et al. 2017; Muhammad et al. 2020; Thorp et al. 2020)
Hydrophobicity	Increased hydrophobicity enhances opsonization, cellular internalization, induces danger signal pathways, stimulates immune cells and innate and adaptive immune responses	(Patel et al. 2015; Liu et al. 2017; Muhammad et al. 2020)
Composition	Polymeric NP can carry a wide range of molecules and can control release of their load thus are potent, versatile therapeutics but are cytotoxic and often lack degradability Lipid NP are biocompatible and less cytotoxic but attract negatively-charged biomolecules forming corona, may be pro-inflammatory Peptides enhance transfection efficiency but are unstable *in vivo* due to susceptibility to protease degradation hence premature release of payload	(Chen et al. 2017; Thorp et al. 2020)

Uptake of NP is affected by size, geometry, stiffness, and surface charge (Moghimi and Szebeni 2003; Geiser 2010; Andon et al. 2017; Boraschi et al. 2017; Costa et al. 2018). The size of an NP plays a substantial role in the way they are perceived: size affects active cellular internalization, dictates their uptake route, and determines their ability to be passively internalized. In general, smaller particles exhibit more cytotoxicity than larger ones as they have a larger surface-to-volume ratio which increases their ability to interact with immune system components (Merkel et al. 2012; Kononenko et al. 2015; Liu et al. 2017). Spherical NP are rapidly internalized by macrophages (Patel et al. 2015; Boraschi et al. 2017); non-spherical shapes require more cytoskeletal rearrangements and are less likely to be taken up. Rigid and stiffer particles are better recognized and internalized by phagocytes, compared with flexible and softer particles, as the latter deform during phagocytosis, rendering it an energy-inefficient process (Patel et al. 2015; Boraschi et al. 2017; Zhao and Stenzel 2018).

A high cationic surface charge improves the transfection efficiency but can also be cytotoxic. Cationic NP are notorious for being more cytotoxic than anionic or neutral NP; it is thought that this property originates from the presence of negatively-charged sialic acid on cell surfaces, which interacts with cationic material (Kononenko et al. 2015). High surface charges can stimulate pro-inflammatory T_H_1 responses (Lam et al. 2012; Lonez et al. 2012; Koppolu and Zaharoff 2013; Getts et al. 2015; Barillet et al. 2019). Koppolu and Zaharoff (2013) showed that cationic chitosan NP encapsulating FITC-BSA caused DC activation and subsequent release of pro-inflammatory cytokines interleukin (IL)-1β, IL-6, MCP-1, MIP-1α, as well as tumor necrosis factor (TNF)-α. Similarly, Barillet et al. (2019) demonstrated that cationic polymeric NP caused monocyte-derived DC activation and secretion of the pro-inflammatory cytokines IL-1β, IL-6, IL-8, and TNFα. The cell membrane senses the cationic charges and triggers intracellular pathways that activate pro-apoptotic or pro-inflammatory reactions (Lonez et al. 2012). High surface charges pose a number of other problems for nano carrier vectors. Nucleic acids can associate too strongly with the vector, and consequently they fail to dissociate from the nano-carrier (Rehman et al. 2013). Serum proteins can bind nonspecifically to the vector and enhance its removal from circulation (Pozo-Rodríguez et al. 2009; Hwang et al. 2015).

### Re-designing NP to evade immunologic responses

Re-design and re-formulation of non-viral delivery systems can enhance the efficacy of gene delivery and reduce adverse immune responses. When treating chronic lung diseases such as asthma and COPD, residence time in the lungs should be maximized to allow local therapeutic effects to occur (Patel et al. 2015). Absorption into the systemic circulation should be avoided, as inhaled NP could be quickly destroyed by nucleases and the reticuloendothelial system (RES) (El-Sherbiny et al. 2015; Ngan and Asmawi 2018). The physicochemical properties themselves can be fine-tuned or the surface of the NP functionalized to evade interaction with immune cells. A promising avenue is to re-design NP to mimic host cells, rendering them immunotolerant.

### Fine-tuning physicochemical properties

NP can be fabricated into different sizes and shapes, and to bear surface charges, which assist in passive evasion of recognition by the host immune system. Aerosolized NP should be designed to have an optimal size that allows them to penetrate and sediment into lower airways lung while avoiding recognition and uptake by innate immune cells. The optimal size for phagocytosis is between 1 and 5 µm (Doshi and Mitragotri 2010; Gustafson et al. 2015). To avoid interaction with, attachment to, and uptake by macrophages, NP should have a size < 1 µm or > 6 µm to increase their residence time in the lungs (Patel et al. 2015).

High surface charge densities, whether they are positive or negative charges, enhance internalization by phagocytes: nanomedicines should therefore have a relatively neutral surface charge. This can be achieved by using zwitterionic or ionizable materials, such as ionizable cationic lipids (Gustafson et al. 2015; Patel et al. 2015). Since soft, flexible and easily-deformed NP are less energetically favorable, and thus less likely to be phagocytosed, NP should be formulated with lipids integrated with cholesterol into the outer layer to increase their fluidity and decrease their rigidity (Patel et al. 2015; Boraschi et al. 2017).

Shapes requiring more actin remodeling are usually less prone to internalization by phagocytic cells, as this would require more energy (Sharma et al. 2010; Boraschi et al. 2017). Independent of their material composition, changing the shape of NP from spherical to non-spherical shapes renders them more resistant to phagocytosis by AM phagocytosis (Champion and Mitragotri 2009; Sharma et al. 2010; Braakhuis et al. 2014; Andon et al. 2017; Boraschi et al. 2017). Shapes like disks, cylinders, and rod-like particles have low curvatures; as such, while these particles can still attach to AM, they are less efficiently internalized because they are not energetically-favorable for membrane wrapping (El-Sherbiny et al. 2015; Lee et al. 2015; Patel et al. 2015). For example, changing the shape of polystyrene particles from spheres to rods or disks reduced cardiopulmonary distress by delaying macrophage clearance following intravenous injection into pigs (Wibroe et al. 2017).

To evade internalization by and activation of AM, NP should adopt a relatively neutral surface charge and have a size outside of the optimal range for phagocytosis (Patel et al. 2015). Some studies have reported that spherical NP have higher rates of internalization and that non-spherical NP evade recognition by macrophages; by this, the latter have longer residence times (Champion and Mitragotri 2009; Sharma et al. 2010; El-Sherbiny et al. 2015; Patel et al. 2015; Boraschi et al. 2017). However, other reports claim that non-spherical NP have higher rates of internalization due to their higher surface areas and geometries similar to that of bacteria that activate macrophages (Doshi and Mitragotri 2010; Thorp et al. 2020).

### NP surface functionalization

Cationic charges can be toxic to cells if the charge density is too high. To reduce immunogenic responses, cationic charges can be masked from the immune system by coating the surface of the NP with PEG. This hydrophilic neutral polymer provides stealth properties, i.e. a water shell forms around the NP to shield the positive charges, reducing adsorption of proteins following entry into the body, resisting opsonization, evading recognition by immune cells and increasing half-life (Ishida et al. 2006; Walkey et al. 2012; Bao et al. 2013; Levine et al. 2013).

On the other hand, PEGylation can prevent positively-charged NP from interacting with negatively-charged membranes, reducing internalization, cargo delivery and endosomal escape (Mansour et al. 2009; Chan et al. 2012; Chen et al. 2016). Administration of PEGylated NP therapeutics can also result in production of anti-PEG IgM. Accelerated blood clearance occurs following administration of a second dose of the therapeutic that activates the classical complement pathway, resulting in rapid degradation of the therapeutic (Ishida et al. 2006; Zhao and Huang 2014; Andon et al. 2017). Production of anti-PEG antibodies is largely dictated by the terminal group of the PEG chain; substituting a methoxy terminal group with a hydroxyl terminal group can mitigate the anti-PEG antibody response, as the hydroxyl group is less antigenic and immunogenic (Sherman et al. 2012; Shimizu et al. 2018). However, the hydroxyl group can activate complement alternative and classical pathways, resulting in the rapid clearance of PEG after administration of a second dose (Shimizu et al. 2018). Alternatively, acid-labile PEG can be used where, at neutral pH, the vector is sterically-stabilized but at acidic pH in endosomes the PEG coat is lost (allowing escape of the genetic material from the endosome) (Chan et al. 2012). In the end, addition of features like acid-labile linkages may present challenges to the development of these delivery systems.

Gene delivery systems can be functionalized to actively target specific cell surface receptors without interfering with the pharmacokinetics of the NP (Mansour et al. 2009; Pozo-Rodríguez et al. 2016). The surface of LNP used to treat cancers has been modified through the attachment of tumor-specific ligands such as transferrin, folic acid, hyaluronic acid, biotin, and anisamide (Huang et al. 2013; Zhao and Huang 2014; Shao et al. 2015; Pozo-Rodríguez et al. 2016). Studies have shown that functionalized LNP achieved increased intracellular nucleic acid delivery and gene expression in cancer cells, in combination with reduced nonspecific cytotoxicity to healthy lung tissue and accumulation in other organs (Taratula et al. 2013; Zhao and Huang 2014; Shao et al. 2015; Pozo-Rodríguez et al. 2016).

### Biomimetics

Biomimetics draw inspiration from nature to produce materials which mimic natural biological processes for application in humans. Synthesizing NP with a surface as complex as the surface of a host cell, incorporating the various membrane proteins and lipids required to be recognized as “self”, is a serious challenge. Instead, to evade the immune system and to increase residence time in the host, investigators have started integrating membrane proteins - such as CD47 derived from red blood cells (RBC) - into the NP formulation (Hu et al. 2011; Fang et al. 2012; Andon et al. 2017). CD47 is a transmembrane protein expressed by all cells and serves as an anti-phagocytic signal; binding to CD172a/signal regulatory protein (SIRP)-α expressed on macrophages and DC prevents internalization and elimination (Rodriguez et al. 2013; Qie et al. 2016; Liu et al. 2017; Fadeel 2019). Polystyrene NP coated with mouse recombinant CD47 incubated with different macrophage subtypes significantly decreased phagocytosis in all macrophages (Qie et al. 2016). In another study, synthetic CD47 peptides were synthesized, conjugated to virus-like particles and injected into mice expressing a CD172a variant compatible with human CD47, which resulted in suppressed phagocytosis, increased circulation time, and drug delivery (Rodriguez et al. 2013).

Other studies have covered NP with RBC membranes to evade recognition by the host immune system (Hu et al. 2011). Poly-(lactic-co-glycolic acid) (PLGA) polymeric NP were covered with RBC membranes containing the associated membrane lipids and proteins through a top-down approach. RBC-membrane-covered NP have a half-life of 39.6 hr (longer than the half-life of 15.8 hr for PEGylated NP) and achieved prolonged drug release *in vivo* in a mouse model. In a different study, adverse cardiopulmonary reactions were reduced after intravenous injection into pigs of sphere-shaped polystyrene particles adhered to RBC (Wibroe et al. 2017).

In a separate study, a different bioinspired therapeutic was synthesized (termed “leuko-some”) by incorporating surface proteins from leukocytic plasma membranes into lipid NP loaded with dexamethasone (Molinaro et al. 2016). These leukosomes were delivered to lipopolysaccharide (LPS)-treated mice, resulting in immune tolerance, increased circulation time, preferential accumulation into the inflamed ear tissue where LPS was injected, and reduced inflammation.

To be used clinically, RBC-membrane-covered NP will need to be compatible with the blood group of the patient or these antigens should be removed during synthesis to avoid inducing immunogenic responses. RBC membranes used to coat the NP could even be derived from the patient receiving the NP to further minimize immune responses (Hu et al. 2011).

Recent studies have explored exosomes as biomimetics to not only increase tropism of NP to tissues but also to increase safety. Exosomes are vesicles with sizes between 30 and 200 nm composed of lipid bilayers which originate in endosomes and are naturally secreted by cells (Khongkow et al. 2019; Liu et al. 2019; Yong et al. 2019). Since the composition of exosomes is similar to that of mammalian cells, they are safe, biocompatible and do not display cytotoxicity (Khongkow et al. 2019; Liu et al. 2019; Yong et al. 2019). Their great biocompatibility, prolonged circulation, stability and low immunogenicity make exosomes attractive as nanocarriers and as biomimetics. Membranes of exosomes are composed of endosomal membrane and plasma membrane proteins that allow them to actively target cells and then be efficiently internalized via endocytosis. Exosome membrane proteins tetraspanin CD9 and CD81 are responsible for membrane fusion and CD55 and CD59 protect exosomes from complement attack and ensure prolonged blood circulation time (Liu et al. 2019).

Exosome-enclosed NP are normally produced by extrusion through nanoporous membranes (Liu et al. 2019; Yong et al. 2019); however, this process may compromise the integrity and stability of the membrane proteins which are crucial for targeted delivery (Yong et al. 2019). Microfluidic sonication has been proposed as an alternative, more effective and quicker process that continuously generates homogenous batches of exosome membrane-coated NP exhibiting a typical spherical core-shell structure (Liu et al. 2019). That study demonstrated that tumor cell-derived exosome-membrane coated PLGA NP have immune-evading abilities due to high expression of CD47 on their membranes as shown by reduced uptake by macrophages and monocytes in *in vitro* and *in vivo* models. Furthermore, by coating NP with exosomes derived from tumor cells, a strong preference and targeting to source tumor cells is exhibited (Liu et al. 2019). Yong et al. (2019) produced exosome-enclosed doxorubicin-loaded porous silicon NP for targeted delivery of chemotherapeutic drugs to tumors. Following intravenous injection, this exosome-biomimetic NP exhibited enhanced extravasation from blood vessels, tumor accumulation, deep tumor penetration and cytotoxicity in total cancer cells and cancer stem cells. These exosome-biomimetic NP produce significant anti-cancer and cancer stem cell killing activity.

To further emphasize the promising application of exosomes, Khongkow et al. (2019) coated gold NP with genetically-modified exosome membranes expressing membrane proteins which enhance penetration through the blood-brain barrier and confer tropism for neuronal cells. Following intravenous injection, gold NP enclosed in genetically-modified exosome membranes (compared to those coated with unmodified exosomes) had superior transport across the blood-brain barrier and increased targeting specificity toward brain cells.

## Conclusions

Gene therapy as a treatment for pulmonary diseases is a promising therapeutic avenue, through the silencing of mutated genes, using anti-sense oligonucleotides (ASO) or RNAi, or the insertion of deleted genes using DNA. Due to the vulnerability of naked nucleic acids in biological fluids, non-viral gene vectors formulated with different materials bearing different physicochemical properties have been designed to shield genetic material with therapeutic purposes from nucleases and to enhance cellular internalization. Investigators have found that NP should be biodegradable to avoid detrimental accumulation in cells and organs, and should adopt a neutral surface charge to avoid adsorption to biological molecules/interactions with RES components. However, investigators still debate the ideal size for NP delivery systems, as their size not only affects surface area but also largely depends on the administration route, the target organ and its environment. The ideal way to deliver therapeutics into the lungs is *via* inhalation as a liquid aerosol or a dry powder formulation; future *in vitro* and *in vivo* studies should investigate optimal inhalable RNA and DNA formulations for gene therapy in the lung. Studies have shown that, in the steady state, the progressively bifurcating architecture of the lung makes it difficult for inhaled therapeutics to deposit and to be retained in the lung long enough to exert therapeutic effects. Other studies have demonstrated that it is important to understand the physiological and anatomical changes in the lung during disease to optimize delivery of the vector to the airways and into target cells.

Literature on the cytotoxicity and immunogenicity of inhaled NP as gene delivery vectors is slowly evolving. We do not know what happens *in vivo*: whether NP degrade or agglomerate, whether they interact with biomolecules and, if so, which ones. The minimum toxic dosage of NP requires further investigation, along with an examination of which components, i.e. the shell, the core or the nucleic acid - might contribute to toxicity. We also require more detailed knowledge on how the host immune response to NP changes over time and to chronic dosing where required, and whether changing one property of the NP changes another, such that a harmless NP might become more harmful. We know that changing the physical properties of NP (size, charge, material, shape, hydrophobicity) alters how they interact with the lung environment. In disease, the local immune system in the lung and the lung environment is dysregulated, i.e. over- or underactive, so it is essential to understand how the pulmonary immune system interacts with and responds to inhaled gene therapeutics in a given disease state. Studying the interaction of inhaled therapeutic NP with the lung immune system *in vivo* will help optimize and maximize delivery of nucleic acid therapeutics and assist in redesign to create optimal NP formulations that could potentially evade immune cells whilst enhancing gene delivery to target cells.
